# Continually improving standards of care: The UK Renal Registry as a translational public health tool

**DOI:** 10.1007/s00467-017-3688-2

**Published:** 2017-06-22

**Authors:** Lucy A. Plumb, Alexander J. Hamilton, Carol D. Inward, Yoav Ben-Shlomo, Fergus J. Caskey

**Affiliations:** 1 The UK Renal Registry, Learning & Research Building, Southmead Hospital, Bristol, UK; 2 School of Social and Community Medicine, University of Bristol, Canynge Hall, 39 Whatley Road, Bristol, BS8 2PS UK; 3 University Hospitals Bristol NHS Foundation Trust, Bristol, UK; 4 The Richard Bright Renal Unit, Southmead Hospital, North Bristol NHS Trust, Bristol, UK

**Keywords:** Registry, Kidney disease, Translational research, Standards

## Abstract

A disease registry uses observational study methods to collect defined data on patients with a particular condition for a predetermined purpose. By providing comprehensive standardised data on patients with kidney disease, renal registries aim to provide a ‘real world’ representation of practice patterns, treatment and patient outcomes that may not be captured accurately by other methods, including randomised controlled trials. Additionally, using registries to measure variations in outcomes and audit care against standards is crucial to understanding how to improve quality of care for patients in an efficacious and cost-effective manner. Registries also have the potential to be a powerful scientific tool that can monitor and support the translational process between research and routine clinical practice, although their limitations must be borne in mind. In this review, we describe the role of the UK Renal Registry as a tool to support translational research. We describe its involvement across each stage of the translational pathway: from hypothesis generation, study design and data collection, to reporting of long-term outcomes and quality improvement initiatives. Furthermore we explore how this role may bring about improvements in care for adults and children with kidney disease.

## Introduction

A disease registry is an organised system that uses observational study methods to capture defined data on patients with a particular condition for a predetermined purpose [1]. Registries provide a comprehensive ‘real world’ representation of practice patterns, treatment and patient outcomes that may not be accurately captured by other methods, including randomised controlled trials (RCTs) [2]. The oldest disease-specific registry dates from 1856, and recent literature estimates that there are over 200 such registries in the UK alone [3].

Registries have the potential to help improve patient care by supporting the translation of research into clinical practice. By collecting longitudinal data, registries provide epidemiological information regarding the natural history and burden of disease for the population examined, which may help to inform research and the development of clinical trials. From a public health perspective, registries can monitor trends in disease and treatment outcomes, and they have the ability to assess the impact of new interventions, as well as audit attainment of National guidance [4]. This is particularly relevant for paediatric chronic kidney disease (CKD), where a small population of patients require complex and costly treatments, and in whom certain outcomes may not be evident for long periods of time.

Many renal registries exist globally that focus on patients with end-stage renal disease (ESRD) requiring renal replacement therapy (RRT; dialysis or renal transplantation). A recent systematic review identified 48 such registries around the world, although more have since been established [5]. Others, however, focus on disease subgroups (e.g. The PodoNet Consortium for patients with steroid-resistant nephrotic syndrome, www.podonet.org) or specific treatments (e.g. the International Paediatric Dialysis Network, www.pedpd.org). The UK Renal Registry (UKRR) was founded in 1995 to improve equity of access to and quality of care for patients receiving treatment for ESRD. Since its inception, it has adapted to changing healthcare, governance, research and technological requirements to become a pioneering registry with regards to centre-specific reports, health services research, quality improvement initiatives and more recently, access to near real-time patient data [6]. In this review, we seek to outline and describe the structure and roles of the UKRR with case examples and to highlight ways in which these contribute to improving care standards across a translational research spectrum.

## Translational research

Increasingly, there is frustration at the delays in effective translation of research findings into practice: “that knowledge and new treatments reach patients for whom they were intended and are implemented appropriately” [7, 8]. It has been recommended that investment in this area will create an efficient and cost-effective research process whilst driving improvements in health policy [9, 10]. This is particularly vital for the field of nephrology, where a significant proportion of healthcare expenditure is spent on a relatively small number of patients [11].

**Fig. 1:**
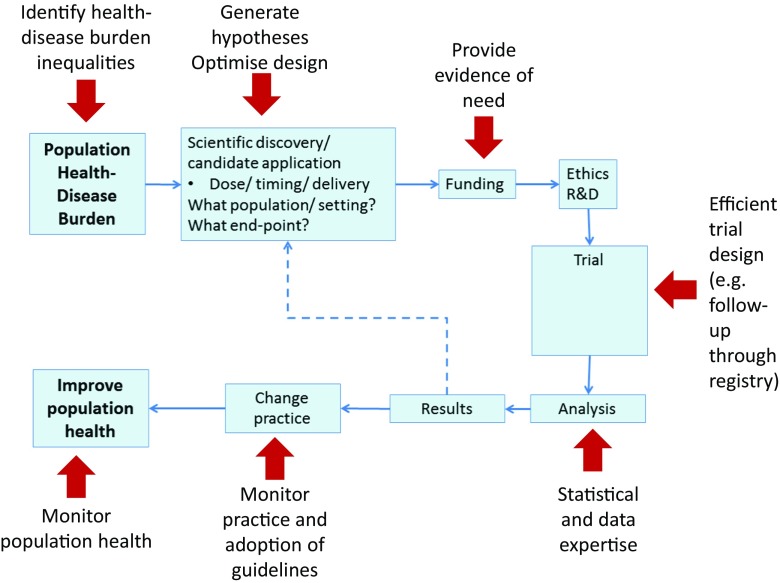
Potential roles for renal registries within the translational health research pathway.* Red arrows* Aspects of pathway where renal registries can have an impact.* R&D* Research and Development. Reproduced with permission from the UK Renal Registry

Two key translational gaps have been identified: (1) the transition of ideas from basic and clinical research into the development of new products and approaches to treatment and (2) the implementation of these products or approaches into clinical practice [12]. This second translational gap has been deemed a priority in the field of nephrology [13]. From identifying causal associations to supporting trial design and analysis, the UKRR and translational epidemiology play a crucial role in this research process and in knowledge synthesis [14, 15] (Fig. 1).

## UKRR development and structure

Before the UKRR, manually collected data was gathered by the European Renal Association and European Dialysis and Transplant Association (ERA-EDTA; previously named the EDTA Registry), for England, Wales, Scotland and Northern Ireland. In 1991 the Scottish Renal Registry (SRR) was established [16], followed by the UKRR in 1995. From the outset, the aim of the UKRR was to provide a resource to monitor UK patient care and to improve equity of access to RRT. Its approach was to establish a database with the capability to extract data electronically from health records in each renal unit. This was followed, in 1997, by the development of a British Association For Paediatric Nephrology (BAPN) paediatric registry database which later amalgamated with the UKRR in 2009. Data from the paediatric renal units in Scotland comes direct to the BAPN database at the UKRR. Currently, work is ongoing to fully integrate paediatric data into the main UKRR database so that the care of patients can be followed seamlessly from childhood to adulthood.

Today, the UKRR is a readily accessible source of patient-level data for researchers, clinicians, health commissioners and the public. It extracts, analyses and reports data from 71 adult and 13 paediatric renal centres, with cleaned, validated data from the 11 Scottish adult renal units coming via the SRR. It can link to external databases, including Hospital Episode Statistics (HES; for hospital admissions), the Office of National Statistics (ONS; for mortality) and Public Health England (for reportable infections, like methicillin-resistant/methicillin-sensitive* Staphylococcus aureus*,* Clostridium difficile* and* Escherichia coli* bacteraemia), thus substantially enhancing its use in audit and research. Data collection is now extending beyond RRT however. Following pilot work [17], data collection has begun for acute dialysis and plasma exchange in acute kidney injury, an area not covered by many other renal registries. Since January 2016, data capture also includes patients with an estimated glomerular filtration rate (eGFR) of <30 ml/min/1.73m^2^. These data will enable a novel comprehensive assessment of the pre-dialysis cohort, including preparation for dialysis and quality of care for people with ESRD who choose not to start RRT.

## Roles of the UKRR across the translational research spectrum

### Providing evidence of the need for research

The collection and interpretation of longitudinal data by renal registries enable users to understand trends in disease incidence or prevalence among different patient populations [18]. By gathering data that is centre- and region-specific, the UKRR is able to highlight disparities in renal outcomes across the UK that require further investigation and health service investment.

Registries can identify novel associations or gaps in knowledge that may lead to the generation of hypotheses and, where appropriate, the subsequent development of funding applications for primary research to explore these in detail and develop interventions. An example of this role is the recently awarded National Institute of Health Research (NIHR) fellowship: ‘Why do children with chronic kidney disease present late to specialist services?’ (NIHR DRF 2016–09-055). This study, designed to examine whether modifiable factors exist for children who present to specialists within a 90-day window from commencing RRT, was developed following a UKRR report describing the cross-sectional burden among the UK paediatric cohort [19].

Registries have historically focused on process measures (e.g. treatment modality, treatment duration/ frequency) and routine clinical outcomes (e.g. anaemia and bone mineral metabolism), with little or no data covering the life outcomes that are important to paediatric patients and their families, such as educational attainment, future prospects and psychological health. To address this, primary research can be embedded within the UKRR to identify key psycho-social variables which could be incorporated into routine data collection. The Surveying People Experiencing young Adult Kidney failure (SPEAK) study [20] sets out to achieve this. By collecting socio-demographic, psychological health and lifestyle data and linking these to UKRR data, SPEAK seeks to understand whether certain clinical variables, including age of disease onset, are associated with poorer quality of life outcomes in young adults. It is expected that the SPEAK study will be powered to identify ‘at risk’ populations that may benefit from targeted interventions.

### Designing studies and data collection

While RCTs have previously been assigned the highest grading of evidence [21], they are often expensive, time-consuming and, due to strict inclusion criteria, have limited external validity (generalizability) to wider patient populations. In addition, for rare subgroups of paediatric kidney disease, small numbers and long follow-up periods often preclude their use. Observational data gathered from renal registries can provide an effective alternative for monitoring patient disease patterns and trends. They can also be used to compare treatment efficacy, although internal validity is limited due to the lack of randomization [22]. Registries may guide development of methodology in observational studies, for example by providing data on the frequency of exposure for any future case–control or cohort studies (where exposure is measured), aiding the calculation of study power.

Where feasible, registries can support RCT development by identifying suitable patients for recruitment and by monitoring long-term outcomes for the studied population. More recently, high-quality registries have been used as the platform from which to recruit, randomise and follow up patients, leading to the emergence of the so-called ‘registry-based RCT’. Compared with conventional RCTs, registry-based RCTs are low in cost and have generally less stringent eligibility criteria, which, together with potential high levels of patient follow-up, may enhance generalizability and provide a more realistic depiction of the impact of treatment [23, 24].

The UKRR has recently been working with the Cambridge Clinical Trials unit to contribute to its first registry-based RCT examining whether ergocalciferol reduces all-cause mortality in adult dialysis patients [25]. The participants will be followed up remotely using registry data and linkage to other clinical datasets, including HES. What is unique to this registry-based trial is that serum calcium levels are being fed back to the clinical trials unit in near real-time, thereby providing an effective feedback mechanism for safety monitoring.

### Monitoring practice–audit and benchmarking

Registries can monitor whether best practice is implemented and clinicians are adhering to updated, evidence-based national guidelines. The UKRR audits data against national standards and ensures that centres are achieving a minimum standard of patient care, with this information made open to scrutiny from patients and the public in annual reports. Examples of this in paediatrics include adherence to National standards set by the National Institute for Clinical Excellence (NICE) and BAPN for anaemia and transplant-recipient blood pressure management, respectively [26, 27]. Data presented by centre must be cautiously interpreted, however, with adjustment for differences in case mix before any conclusion can be drawn on the possible existence of “centre effects” [28]. Centre-level comparisons, both against each other and with national guidelines, allow high-performing centres to be identified and provide insight into unmeasured structural and organisational processes that may subsequently drive quality improvement initiatives in other units.

### Delivering quality improvement

Quality improvement (QI) can be defined as the combined and unceasing efforts of everyone—kidney healthcare professionals, patients with kidney disease and their families, commissioners of kidney services, researchers, and educators—to make changes that will lead to better patient outcomes, better system performance and better professional development [29].

By continuously collecting and comparing outcome data from renal units, renal registries are well placed to underpin quality improvement work. In conjunction with the Health Foundation and Kidney Research UK, the ASSIST-CKD (A programme to Spread eGFR graph Surveillance for the early Identification, Support and Treatment of people with CKD) study is an example of UKRR data being used to provide outcomes and analysis for QI. With the overarching aim of reducing late presentation in the adult CKD population, ASSIST-CKD uses a novel software package embedded in hospital laboratories to generate eGFR graphs from patient results over time, alerting primary care teams of deterioration that may not have been otherwise identified [30]. Routine data collection of incident ESRD patients (including late presentation) is serving as the outcome measure.

Collaboration is key to successfully raising care standards across the country. This is demonstrated by the recently established Kidney Quality Improvement Partnership (KQuIP) initiative (www.thinkkidneys.nhs.uk/kquip). This network of professionals and patients is committed to developing, supporting and sharing quality improvement initiatives with the purpose of improving the care and quality of life for people of all ages with kidney disease. The network aims to do this by embedding quality improvement programmes into clinical practice, by understanding and reducing variation in care and by sharing good practice among renal units.

## International comparisons and collaborations

Data from renal registries can be used to compare patient outcomes at the international level, and the UKRR provides anonymized data to the ERA-EDTA Registry [31] as well as aggregate data to the US Renal Data System (USRDS) [32]. Such global collaborations enable clinicians and researchers to study rare disease outcomes in larger populations and to identify variations in outcomes that may occur due to differing practice patterns between countries. Collaborative initiatives such as the European Reference Network (ERN) for kidney disease will also help optimise the development and implementation of innovative treatments, whilst providing a focal point within the European community for training, research and information sharing [33].

Efficient sharing of information among countries, however, requires collaboration between renal registries to standardise data items and data sets. A good example of this is the ERA-EDTA Registry’s ‘QUality European STudies’ (QUEST) initiative, which has supported the development of standardised datasets and increased availability of data on clinical performance indicators alongside increased access to automated data collection [34].

## UKRR activities beyond audit and research

### PatientView

**Fig. 2:**
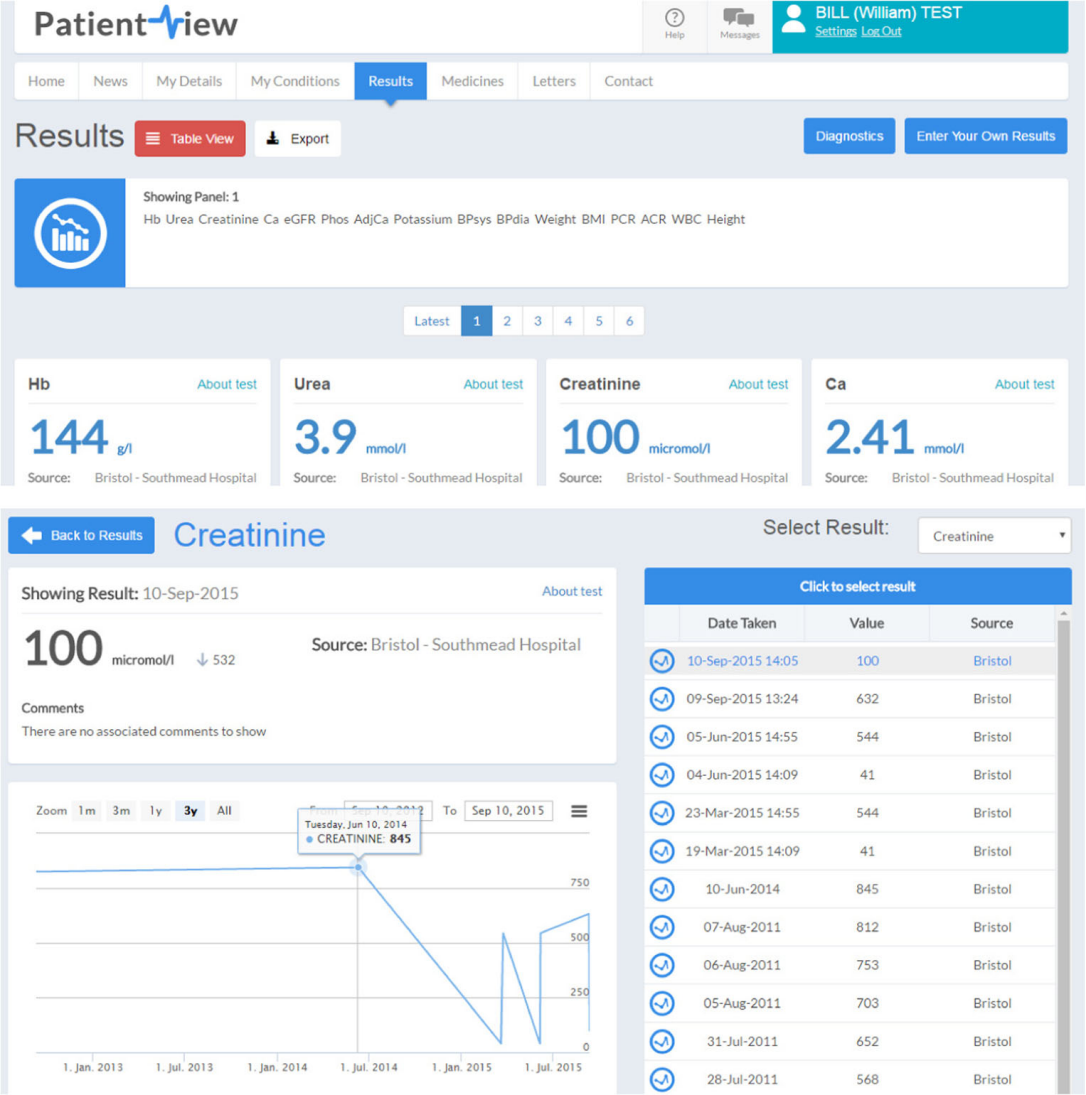
PatientView example screens (adult test patient). Reproduced with permission from the UK Renal Registry

PatientView (PV) is a web-based system that provides patients and clinicians with rapid access to local laboratory results, which are uploaded daily (Fig. 2). In addition, patients can view information regarding their diagnosis and treatment; read clinic letters; enter personal data (such as blood pressure or weight) from their own records. It was established in 2004 by the Renal Information Exchange Group (RIXG) and is now available in 90% of UK nephrology centres, funded by contributions from renal centres in England, Wales and Northern Ireland and by the Government of Scotland. Patient users report satisfaction with the tool and perceive it to be helpful in managing their condition [35]. Furthermore, its ability to contemporaneously collect data linked to the UKRR and National Registry of Rare Kidney Diseases (RaDaR) is crucial to supporting rare disease research and the development of novel registry-based trials in the UK.

### The National Registry of Rare Kidney Diseases

Informative clinical data is a necessary step to improving the treatment and outcomes of patients with rare diseases. Embedded in the UKRR framework lies the National Registry of Rare Kidney Diseases, which was established in 2011 at the request of the Renal Association and BAPN following UK and EU recommendations to improve access to high-quality care for patients with rare diseases [36, 37].

In partnership with patients, RaDaR aims to facilitate research and improve the care of patients with rare kidney diseases. It is a comprehensive clinical database that captures both generic and disease-specific information on patients from a growing list of diseases (www.rarerenal.org), the most recent being fibromuscular dysplasia and Fabry disease [38]. This database was developed by rare disease experts and patients to develop care standards and research opportunities. Careful characterisation of well-defined patients has supported the development of National Studies into rare disease subgroups (e.g. the National Study of Nephrotic Syndrome, NephroS study) as well as the reporting of novel genotype–phenotype correlations [39, 40]. Long-term surveillance will enable outcomes into adulthood to be described. Grant applications are also more likely to succeed when accurate estimates of patient cohorts are known and patients have already consented to being approached directly about research opportunities. Work is also underway to develop an international version of RaDaR, which will serve to further increase our knowledge of rare renal disease. Recruitment targets set for the NIHR have been amended upward from 10,000 to 25,000, reflecting the success of the rare disease initiative.

In conjunction with PV, RaDaR empowers patients to enter personal information, including patient-reported quality of life and outcome measures, into their clinical records. By operating within the UKRR, PV and RaDaR benefit from the existing governance infrastructure for automated data collection, as well as its technical expertise. Ongoing research into rare renal disease also supports evolution of the UKRR dataset as our understanding of disease determinants improves. Future developments, including a national kidney biobank, may be supported by the RaDaR platform, enabling linkage of patient’s clinical information with biological data. Together with the UKRR, PV and RaDaR offer a sustainable resource of longitudinal information on rare diseases that may help drive forward improvements in care pathways, access to services and research into better treatments.

## Limitations of registries and the UKRR

Limitations to registries exist and must be considered when using data. Registries can be expensive and time-consuming to establish and maintain. They are also only as accurate as the data entered at each renal unit and so rely on front-line clinicians to ensure data returns are correct and complete. For example, in the UK, data completeness for comorbidity at the start of RRT remains at an undesirable level (29/62 of UK adult centres have <75% data completeness). This state of affairs limits the UKRR’s ability to directly adjust for case mix [6], which may be particularly problematic when the presence of comorbidity may be clinically relevant to ESRD outcomes [41, 42]. Missing data may introduce bias, and imputation methods cannot fully overcome this problem. Linkage to other routine databases has been successfully used in large observational studies to address this problem [43] and is being explored within the UKRR framework. For the paediatric dataset, relatively small numbers of patients can restrict the statistical power achievable in observational studies, but this can be improved by sharing data with other countries through, for example, the European Society for Paediatric Nephrology (ESPN) ERA-EDTA Registry. Furthermore, observational studies generated using registry data are generally hypothesis generating and can only rarely demonstrate causality (e.g. assessing a natural experiment) [4]. Despite the use of statistical methods to adjust for observed confounders, there is always the possibility that unmeasured confounders may have generated false associations. Finally, laboratory parameters obtained from renal centres may demonstrate a high degree of variability for a given test, although this effect is likely to be small [42].

## Future directions

**Fig. 3:**
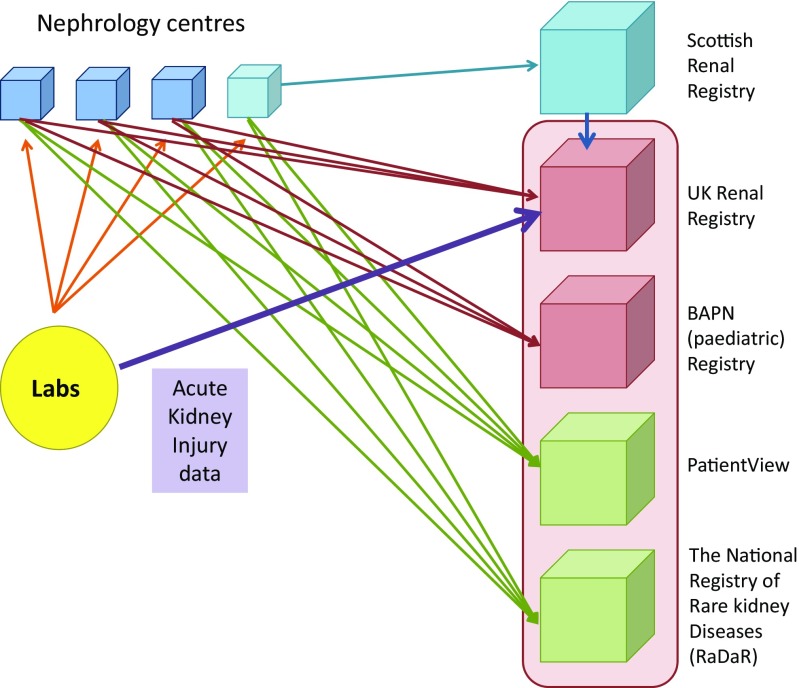
Flow diagram of data collection within the UK Renal Data Collaboration (UKRDC).* BAPN* British Association For Paediatric Nephrology

To continue supporting improvements in the care of people with kidney disease, registries must have the capacity to evolve—both in terms of their technological infrastructure as well as their involvement in translational health research and development of health policy. Recognising this, the UK Renal Data Collaboration (UKRDC) was established in 2013. The UKRDC seeks to enable data from electronic health records to be standardised for the purposes of all member organisations (Fig. 3), supporting the move towards paperless records and ‘streamlining’ data use to improve efficiency for renal units. It will provide a more dynamic platform from which datasets can be added to or amended, allowing data collection to evolve as our knowledge of renal disease advances [44]. It has already supported the running of an RCT, SIMPLIFIED (discussed above), with contemporaneous feedback for certain adverse events monitoring and has the potential to support the collection of patient-reported outcome measures via PV in national data collection.

The aim of the recent UK Renal Research Strategy (2016) is to support the improvement of renal care through research [13]. Developed with professional and patient bodies, its recommendations include increased engagement of professionals and public with research; capitalisation of the full spectrum of research opportunities for maximum benefit to patient care; establishment of a multi-disciplinary and collaborative approach to research; and supporting the training of future researchers. We believe that the UKRR can play an important role in bringing each of these aims to fruition in a sustainable and cost-effective manner.

## References

[1] Glicklich R, Dreyer N, Leavy M (eds) (2014) Registries for evaluating patient outcomes: a user’s guide, 3rd edn. Agency for Healthcare Research and Quality, Rockville. http://www.effectivehealthcare.ahrq.gov/registries-guide-3.cfmdoi: AHRQ Publication No. 07-EHC001–1 24945055

[2] Gitt AK, Bueno H, Danchin N, Fox K, Hochadel M, Kearney P, Maggioni AP, Opolski G, Seabra-Gomes R, Weidinger F (2010). The role of cardiac registries in evidence-based medicine. Eur Heart J.

[3] Newton J, Garner S (2002) Disease registers in England. Institute of Health Sciences, University of Oxford, Oxford

[4] Birnie K, Caskey F, Ben-Shlomo Y, Sterne JAC, Gilg J, Nitsch D, Tomson C (2017). Erythropoiesis-stimulating agent dosing, haemoglobin and ferritin levels in UK haemodialysis patients 2005—13. Nephrol Dial Transplant.

[5] Liu FX, Rutherford P, Smoyer-Tomic K, Prichard S, Laplante S (2015). A global overview of renal registries: a systematic review. BMC Nephrol.

[6] Caskey F, Cullen R (2016). UK renal registry 18th annual report 2015: introduction. Nephron.

[7] Sung NS, Crowley WF, Genel M, Salber P, Sandy L, Sherwood LM, Johnson SB, Catanese V, Tilson H, Getz K, Larson EL, Scheinberg D, Reece EA, Slavkin H, Dobs A, Grebb J, Martinez RA, Korn A, Rimoin D (2003). Central challenges facing the National Clinical Research Enterprise. JAMA.

[8] Woolf SH (2008). The meaning of translational research and why it matters. J Am Med Acad.

[9] Editorial (2006), Reorganising research in the UK. Lancet 368:2105 10.1016/S0140-6736(06)69839-5 17174688

[10] Van Der Veer SN, Van Biesen W, Couchoud C, Tomson CRV, Jager KJ (2014). Measuring the quality of renal care: things to keep in mind when selecting and using quality indicators. Nephrol Dial Transplant.

[11] De Vecchi AF, Dratwa M, Wiedemann ME (1999). Healthcare systems and end-stage renal disease (ESRD) therapies—an international review: costs and reimbursement / funding of ESRD therapies. Nephrol Dial Transplant.

[12] Cooksey D (2006) A review of UK health research funding. The Stationary Office, London. hm-treasury.gov.uk

[13] Frankl F, Coward R, Gallagher H, Hilton R, Loud F, Modi K, Ormandy P, Woolf A (2006) UK renal research strategy. UK Kidney Res Consortium. https://www.kidneyresearchuk.org/research/ukkrc

[14] Ogilvie D, Craig P, Griffin S, Macintyre S, Wareham NJ (2009). A translational framework for public health research. BMC Public Health.

[15] Khoury MJ, Gwinn M, Ioannidis JPA (2010). The emergence of translational epidemiology: from scientific discovery to population health impact. Am J Epidemiol.

[16] Scottish Renal Registry. http://www.srr.scot.nhs.uk/. Accessed 17 Jan 2017

[17] Selby NM, Casula A, Lamming L, Mohammed M, Caskey F (2016). Design and rationale of “tackling acute kidney injury”, a multicentre quality improvement study. Nephron.

[18] Hamilton AJ, Casula A, Ben-Shlomo Y, Caskey FJ, Inward CD (2016). Clinical epidemiology, treatment & survival of young adults starting renal replacement therapy in the UK using 15 years of UK renal registry data. Nephrol Dial Transplant.

[19] Pruthi R, Casula A, Inward C, Roderick P, Sinha MD (2016). Early requirement for RRT in children at presentation in the United Kingdom: association with transplantation and survival. Clin J Am Soc Nephrol.

[20] Hamilton A (2014) Surveying people Experiencing young Adult Kidney failure (SPEAK) study. https://www.renalreg.org/projects/speak. Accessed 5 Jan 2017

[21] Concato J, Shah N, Horwitz RI (2000). Randomized, controlled trials, observational studies and the hierarchy of research designs. N Engl J Med.

[22] Lauer M, D’Agostino R (2010). The randomized registry trial-the next disruptive technology in clinical research?. N Engl J Med.

[23] Concato J, Horwitz RI (2004). Commentary: beyond randomised versus observational studies. Lancet.

[24] Li G, Sajobi TT, Menon BK, Korngut L, Lowerison M, James M, Wilton SB, Williamson T, Gill S, Drogos LL, Smith EE, Vohra S, Hill MD, Thabane L (2016). Registry-based randomized controlled trials-what are the advantages, challenges, and areas for future research?. J Clin Epidemiol.

[25] Hiemstra TF (2016) Survival Improvement with Cholecalciferol in Patients on Dialysis the SIMPLIFIED registry trial. National Institute for Health Research. https://www.journalslibrary.nihr.ac.uk/programmes/hta/1449127/#/

[26] NICE clinical guideline NG8 (2015) Anaemia management in people with chronic kidney disease. National Institute for Health and Clinical Excellence, London

[27] Sinha MD (2011). BAPN Standards for Hypertension in Paediatric Renal Transplant Recipients. Website: http://www.renal.org/docs/default-source/special-interest-groups/bapn/clinical-standards/bapn-standards-for-hypertension-in-renal-transplant-recipients.pdf?sfvrsn=2. Accessed 18 May 2017

[28] Hodsman A, Ben-Shlomo Y, Roderick P, Tomson CRV (2011). The “Centre effect” in nephrology: what do differences between nephrology centres tell us about clinical performance in patient management?. Nephron Clin Pract.

[29] Batalden PB, Davidoff F (2007). What is “quality improvement” and how can it transform healthcare?. Qual Saf Health Care.

[30] Gallagher H, Methven S, Casula A, Thomas N, Tomson CRV, Caskey FJ, Rose T, Waters SJ, Kennedy D, Dawnay A, Cassidy M, Fluck R, Rayner HC, Nation M (2017). A programme to spread eGFR graph surveillance for the early identification, support and treatment of people with progressive chronic kidney disease (ASSIST-CKD): protocol for the stepped wedge implementation and evaluation of an intervention to reduce late presentation for renal replacement therapy. BMC Nephrol.

[31] ERA-EDTA Registry (2016) ERA-EDTA Registry Annual Report 2014. Academic Medical Center, Department of Medical Informatics, Amsterdam

[32] US Renal Data System (2016) USRDS 2016 Annual Data Report: End stage renal disease (ESRD) in the United States. Chapter 13: International Comparisons. National Institutes of Health, National Institute of Diabetes and Digestive and Kidney Diseases, Bethesda, pp 291–334

[33] NHS England (2017) NHS England: European Reference Networks. https://www.england.nhs.uk/commissioning/spec-services/highly-spec-services/ern/. Accessed 14 Apr 2017

[34] Jager KJ, Zoccali C (2005). QUality European STudies (QUEST)—a step forward in the quality of RRT care. Nephrol Dial Transplant.

[35] Woywodt A, Vythelingum K, Rayner S, Anderton J, Ahmed A (2014). Single-centre experience with renal PatientView, a web-based system that provides patients with access to their laboratory results. J Nephrol.

[36] Simerka P (2009). EU Council recommendation—action in the field of rare diseases (8th June 2009). Off J Eur Union.

[37] Donaldson L (2010) 2009 Annual report of the chief medical officer. London Department of Health, London

[38] Dillon M (2016) RaDaR Newsletter Autumn 2016- Issue Seven. http://rarerenal.org/wp-content/uploads/2016/10/RaDaR-Newsletter-Autumn-2016.pdf. Accessed 11 Apr 2017

[39] McCarthy HJ, Bierzynska A, Wherlock M, Ognjanovic M, Kerecuk L, Hegde S, Feather S, Gilbert RD, Krischock L, Jones C, Sinha MD, Webb NJA, Christian M, Williams MM, Marks S, Koziell A, Welsh GI, Saleem MA (2013). Simultaneous sequencing of 24 genes associated with steroid-resistant nephrotic syndrome. Clin J Am Soc Nephrol.

[40] Wong E, Marchbank K, Pappworth I, Walters R, Lomax-Browne H, Harris C, Morgan P, Pickering M, Goodship THJ, Malcomson R, Cook T, Johnson S (2015) MPGN/C3G Rare Disease Group The national study of membranoproliferative glomerulonephritis and C3 glomerulopathy—characterisation of the paediatric cohort. Nephrol Dial Transplant 30 [Suppl_3]:iii19

[41] Jager KJ, Zoccali C (2009). Comorbidity data collection by renal registries—a remaining challenge. Nephrol Dial Transplant.

[42] Harambat J, Bonthuis M, Groothoff JW, Schaefer F, Tizard EJ, Verrina E, van Stralen KJ, Jager KJ (2016). Lessons learned from the ESPN/ERA-EDTA registry. Pediatr Nephrol.

[43] Cornish RP, Tilling K, Boyd A, Davies A, Macleod J (2015). Using linked educational attainment data to reduce bias due to missing outcome data in estimates of the association between the duration of breastfeeding and IQ at 15 years. Int J Epidemiol.

[44] Cullen R (2013) UK Renal Data Collaboration White Paper. UK Renal Data Collaboration. https://www.renalreg.org/projects/the-uk-renal-data-collaboration-ukrdc/

